# Combining autophagy stimulators and cellulose ethers for therapy against prion disease

**DOI:** 10.1080/19336896.2019.1670928

**Published:** 2019-10-03

**Authors:** Basant A. Abdulrahman, Waqas Tahir, Katsumi Doh-Ura, Sabine Gilch, Hermann M. Schatzl

**Affiliations:** a Department of Comparative Biology & Experimental Medicine, Faculty of Veterinary Medicine, University of Calgary, Calgary, Alberta, Canada; b Calgary Prion Research Unit, University of Calgary, Calgary, Alberta, Canada; c Hotchkiss Brain Institute, University of Calgary, Calgary, Alberta, Canada; d Department of Biochemistry and Molecular Biology, Faculty of Pharmacy, Helwan University, Cairo, Egypt; e Department of Neurochemistry, Tohoku University Graduate School of Medicine, Sendai, Japan

**Keywords:** Prion, prion disease, combination therapy, autophagy, AR12, rapamycin, cellulose ethers, TC-5RW, 60SH-50

## Abstract

Prion diseases are fatal transmissible neurodegenerative disorders that affect animals and humans. Prions are proteinaceous infectious particles consisting of a misfolded isoform of the cellular prion protein PrP^C^, termed PrP^Sc^. PrP^Sc^ accumulates in infected neurons due to partial resistance to proteolytic digestion. Using compounds that interfere with the production of PrP^Sc^ or enhance its degradation cure prion infection *in vitro*, but most drugs failed when used to treat prion-infected rodents. In order to synergize the effect of anti-prion drugs, we combined drugs interfering with the generation of PrP^Sc^ with compounds inducing PrP^Sc^ degradation. Here, we tested autophagy stimulators (rapamycin or AR12) and cellulose ether compounds (TC-5RW or 60SH-50) either as single or combination treatment of mice infected with RML prions. Single drug treatments significantly extended the survival compared to the untreated group. As anticipated, also all the combination therapy groups showed extended survival compared to the untreated group, but no combination treatment showed superior effects to 60SH-50 or TC-5RW treatment alone. Unexpectedly, we later found that combining autophagy stimulator and cellulose ether treatment in cultured neuronal cells mitigated the pro-autophagic activity of AR12 and rapamycin, which can in part explain the *in vivo* results. Overall, we show that it is critical to exclude antagonizing drug effects when attempting combination therapy. In addition, we identified AR-12 as a pro-autophagic drug that significantly extends survival of prion-infected mice, has no adverse side effects on the animals used in this study, and can be useful in future studies.

## Introduction

Prion diseases are neurodegenerative disorders in humans and animals that are invariably fatal. Human prion diseases occur in sporadic, genetic, and acquired forms. The autocatalytic conversion of the non-infectious and endogenously expressed isoform PrP^C^ to the infectious isoform PrP^Sc^ is attributed to be the cause of the disease [1,2]. The human forms of prion disease include familial, sporadic, iatrogenic and variant Creutzfeldt-Jakob disease (CJD), Gerstmann-Sträussler-Scheinker syndrome (GSS), and fatal familial insomnia (FFI). Animal forms are scrapie in sheep and goat, bovine spongiform encephalopathy (BSE) in cattle and other species, and chronic wasting disease (CWD) in deer, reindeer, elk and moose [3–7]. Loss of neurons, vacuolation, astrogliosis and microglial activation is the major histopathological findings in prion disease. This results in spongiform degeneration of the central nervous system (CNS), leading to ataxia, behavioural changes and highly progressive loss of intellectual propensity [8,9].

Prions use living cells for propagation by conformational conversion of endogenous PrP^C^, and newly generated PrP^Sc^ undergoes subcellular trafficking events and is exposed to cellular degradation and recycling machineries. Mechanistically, there are many steps in the life cycle of prions which can be used as therapeutic targets, ranging from PrP^C^ substrate, PrP^C^/PrP^Sc^ interaction to lysosomal PrP^Sc^ clearance [10,11]. A huge variety of chemical compounds was tested in vitro and in vivo for anti-prion activities. Historically, PrP^Sc^ was aimed as therapeutic target [12,13], with an emphasis early on lysosomal degradation [14–16]. Another strategy was targeting PrP^C^, by binding to chemical compounds, other ligands or antibodies [11]. Interestingly, PrP^Sc^ itself is a difficult pharmacological target because of its poorly defined structure and the ability to generate different structural conformers that can confound treatment. As a consequence of the *quasi-species* nature of prions [17], prions are sensitive to the development of drug resistance [18–20]. Although combination therapy could be used to create synergies by targeting different steps in the life cycle of prions, this was hardly done for mammalian prions [21].

Autophagy is the natural, regulated mechanism that allows the orderly degradation and recycling of cellular components [22,23]. We and others have established pharmacological induction of autophagic degradation as a potent anti-prion strategy [24–28]. Our recent work demonstrated that AR12 (OSU-03012, PDK1 inhibitor), a novel autophagy-inducing drug, enhanced the degradation of PrP^Sc^ and reduced prion seeding activity in cell lines infected with various prion strains *in vitro* [29]. Here we test the *in vivo* effect of AR12. Additionally, we used the autophagy stimulator and mTOR inhibitor rapamycin which we have shown previously to extend the survival time of prion-infected mice [26].

Recently, cellulose ethers (CEs) have emerged as promising anti-prion compounds. The CE compounds are non-ionic, non-digestible, water soluble and widely used in the pharmaceutical industry as inert additives. They showed remarkable anti-prion effects with only a single subcutaneous injection [30]. Mechanistically, they are thought to inhibit the conversion of PrP^C^ into PrP^Sc^.

Here, we studied the anti-prion effect of the CE compounds TC-5RW and 60SH-50 when combined with the autophagy stimulators AR12 or rapamycin, under the premise that these drugs target different steps and might therefore induce additive anti-prion effects. We tested four different combination groups (AR12, TC-5RW), (AR12, 60SH-50), (rapamycin, TC-5RW) and (rapamycin, 60SH-50) in prion-infected mice. Our results demonstrate that all combination therapy groups significantly extended survival compared to the untreated group. However, no combination treatment showed superior effects to 60SH-50 or TC-5RW treatment alone. To exclude antagonizing drug effects, we revisited these drug combinations in prion-infected cultured cells. These studies showed that combining autophagy stimulators and cellulose ethers significantly alleviated the autophagic activity of AR12 and rapamycin. This could explain the results obtained *in vivo* and shows that it is critical to exclude antagonizing drug effects when attempting combination therapy.

## Results

### Treatment with autophagy stimulators AR12 or rapamycin significantly prolonged the survival of RML infected mice

**Figure 1.**
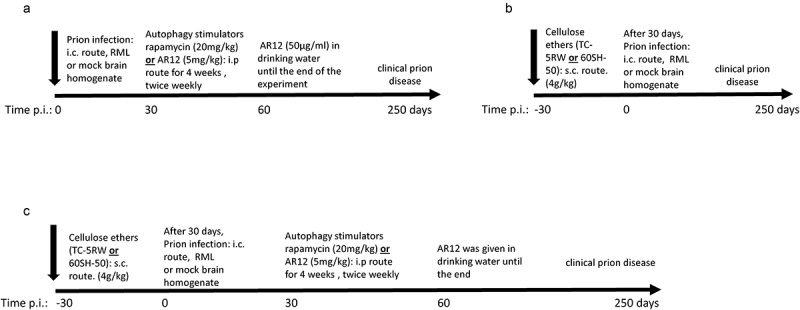
Schematic representation of animal experiments. (a) Scheme of the animal experiments using autophagy inducers AR12 and rapamycin. (b) Schematic representation of animal experiments using cellulose ethers TC-5RW or 60SH-50. (c) Animal experiments using drug combinations of cellulose ethers and autophagy stimulators. FVB mice were inoculated with either RML prions from terminally sick mice or mock brain homogenate.

**Figure 2.**
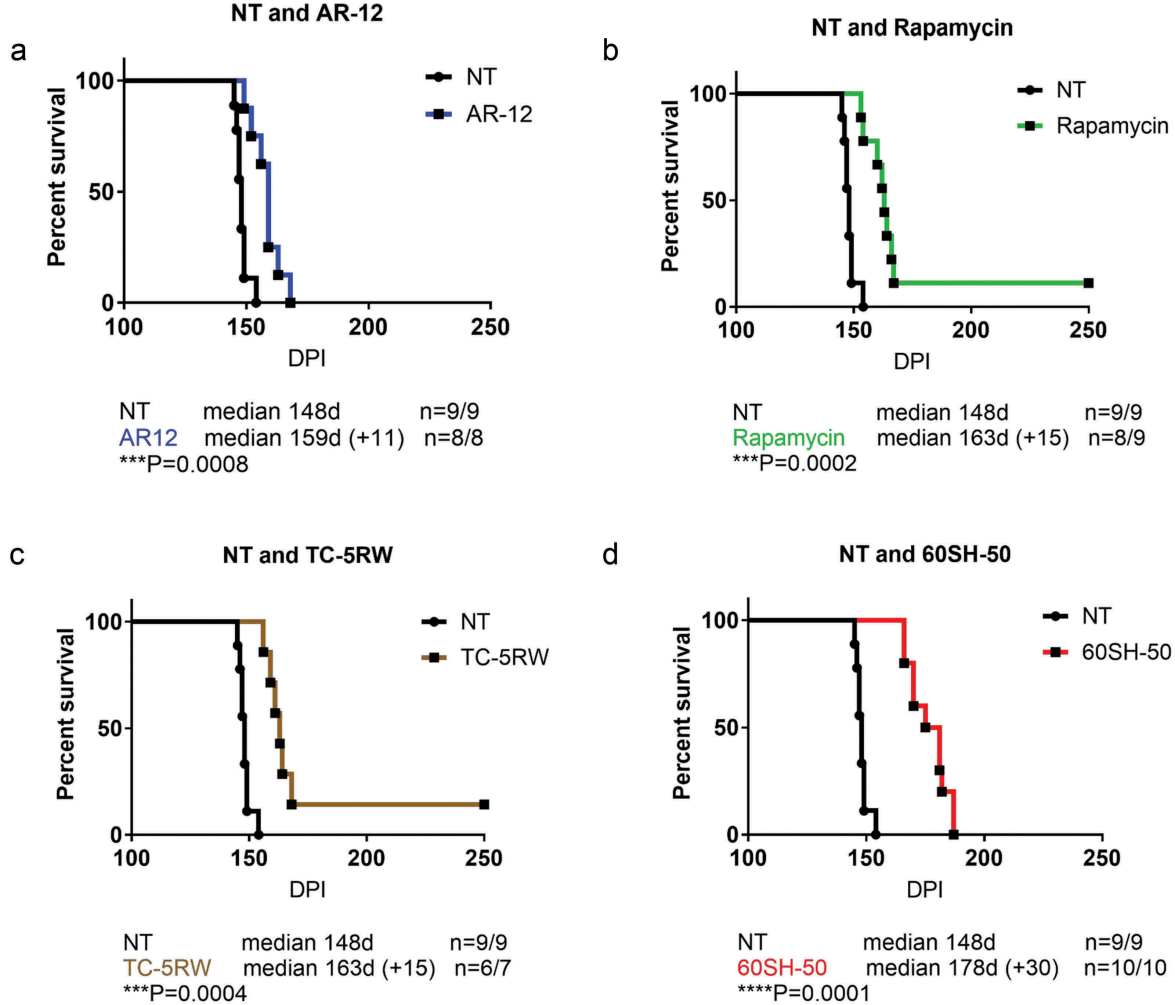
Effect of autophagy stimulators or CE compounds in RML infected FVB mice. Survival analysis of FVB mice intra-cerebrally infected with RML prion was performed. (a) Thirty days after i.c. inoculation mice were treated with AR-12 (5 mg/kg, i.p.) twice per week for 4 weeks, then AR-12 was added to the drinking water (50 µg/ml) until the end of the experiment. (b) Rapamycin treatment started also at 30 dpi. The mice were injected i.p. twice per week (20 mg/kg) for 4 weeks. (c and d) Mice were treated with single subcutaneous injection of either CE-5RW or 60SH-50 at 4g/kg body weight 30 days before i.c. inoculation. Experiments were stopped at 250 dpi.

The role of autophagy in modulating prion disease has been the focus of our research for more than a decade [24–26,31–33]. Here, we examined the effect of treatment with the autophagy inducer AR12 on the survival times of FVB mice infected intra-cerebrally with RML prions. AR12 treatment started 30 days after prion inoculation and was administered through i.p. injection (5 mg/kg body weight) [34] for 4 weeks, twice weekly. After 4 weeks of i.p. treatment, AR12 was administered in drinking water (50 µg/ml) until the experimental endpoint (Figure 1). Our data showed markedly improved survival of the animals treated with AR12 compared with untreated animals (*p = 0.0008*) (Figure 2(a)). In addition, we used rapamycin to compare its effect to AR12. Rapamycin was administered to the animals via i.p. route (20 mg/kg body weight) [35], twice weekly for 4 weeks (Figure 1(a)). Due to the poor solubility of rapamycin in water, we did not continue rapamycin treatment in drinking water. Our results show significant extended survival of the animals treated with rapamycin compared to untreated ones (*p = 0.0002*) (Figure 2(b)). There was no significant difference between the effect of AR12 and rapamycin on the survival time. Mean survival times are displayed in Table 1. Notably, this is the first study to demonstrate the anti-prion effect of AR12 *in vivo*.

**Table 1.** Mean survival time of mice groups.

Animal group	#of mice/group	Treatment	Survival time (DPI) (Mean ± SD)
Mock	9	-	250
Mock-AR12	4	AR12	250
Mock-Rapamycin	4	Rapamycin	250
RML	9	-	148.11 ± 2.57
RML-AR12	8	AR12	158.12 ± 5.96
RML-Rapamycin	9*	Rapamycin	161.83 ± 5.19
RML-5RW	7*	5RW	161.83 ± 4.16
RML-60SH	10	60SH	173.87 ± 6.79
RML-(AR12 + 5RW)	10*	AR12 + 5RW	162.11 ± 3.68
RML-(AR12 + 5RW)	8	AR12 + 60HS	165.75 ± 4.65
RML-(Rapamycin+5RW)	10	Rapamycin+5RW	167 ± 2.74
RML-(Rapamycin+60SH)	8	Rapamycin+60SH	173 ± 9.82

*One animal in the group did not show any prion signs until the end of the experiment and sacrificed at 250 DPI. This one animal is not considered in the calculation of mean since the calculations are only for prion sick mice.

FVB mice were either mock or RML infected. Mock groups were sacrificed at 250 days post mock i.c. inoculation.

AR-12 treatment was started 4 weeks after i.c. inoculation. The drug was I.P. injected for 4 weeks (5mg/ml) then shifted to dissolving the drug in water (50ug/ml, 15,000ug) until the end of the experiment.

Rapamycin treatment started 4 weeks after i.c. inoculation. The drug was I.P. injected for 4 weeks (20mg/ml) only.

Cellulose ethers (5RW and 60SH) were given as a single subcutaneous dose (4g/kg) 4 weeks before i.c. inoculation.

### Treatment with cellulose ethers TC-5RW or 60SH-50 significantly increased survival of RML infected mice

Next, we tested the prophylactic effect of two cellulose ethers, TC-5RW and 60SH-50. Both CE compounds were used in a previous study; however, C57Bl/6 mice were used for these experiments [30]. Given the proposed dependence of CE effects on the genetic background of mice [30], we first wanted to confirm the anti-prion effects of CEs in FVB mice. CEs were administered as a single subcutaneous dose (4g/kg), 30 days before i.c. RML prion inoculation (Figure 1(b)). In line with previous results, we show that treatment with either TC-5RW or 60SH-50 results in a significantly increased survival time (*p = 0.0004*) and p = 0.0001, respectively (Figure 2(c,d); Table 1).

### Combination therapy using autophagy stimulators and cellulose ethers

**Figure 3.**
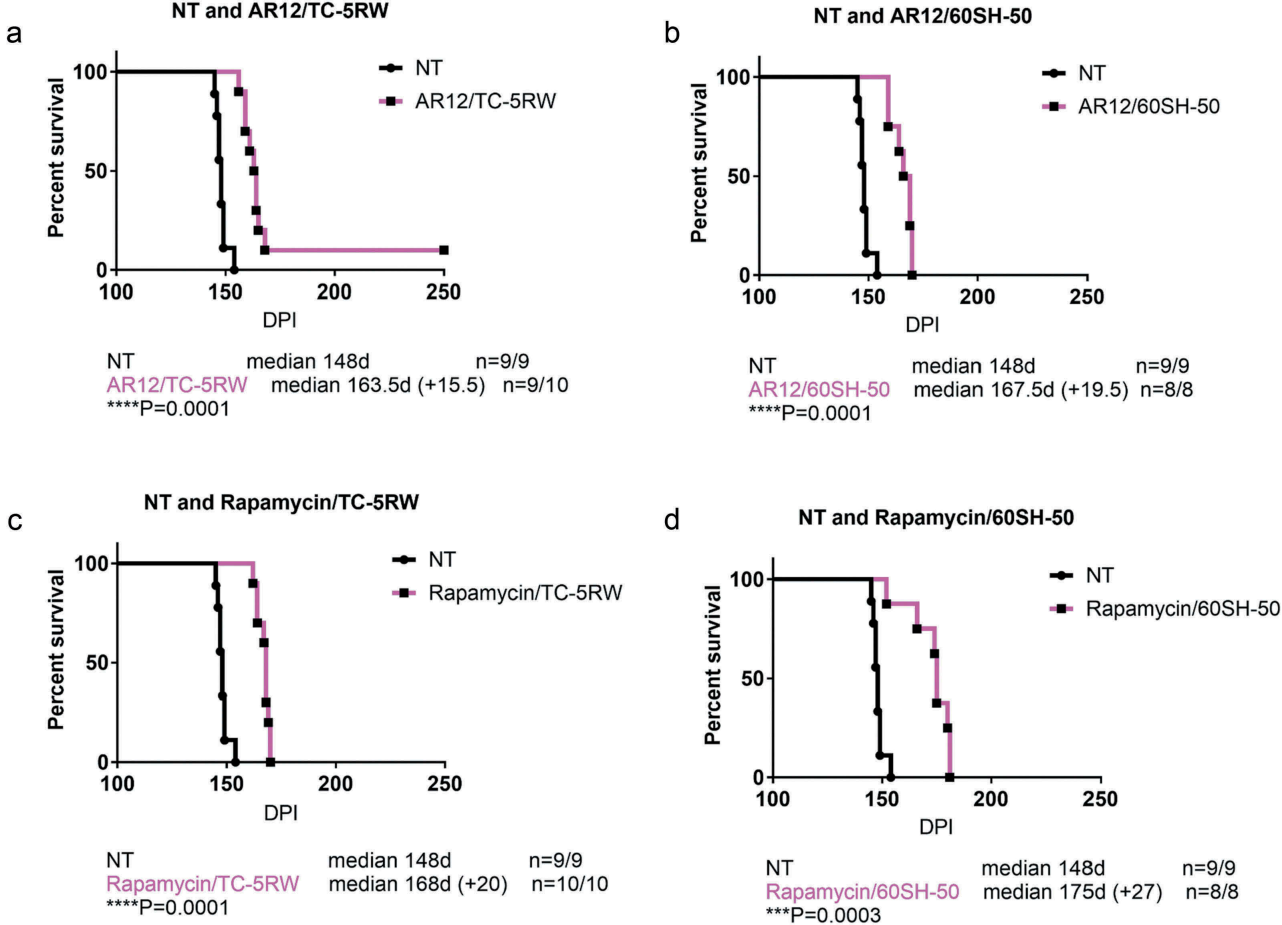
Effect of combined treatment with autophagy stimulators and CEs compared to the mock-treated group. Survival analysis of FVB mice i.c. infected with RML prions. (a) Combination of AR-12 (5 mg/kg i.p twice per week for 4 weeks, then AR-12 was added to the drinking water (50 µg/ml) until the end of the experiment) and single subcutaneous injection of TC-5RW one month before prion inoculation. (b) Combination of AR-12 (treatment as in a) and single subcutaneous injection of 60SH-50 one month before prion inoculation. (c) Combination of rapamycin (starting at 30 dpi, treatment (i.p.) twice per week (20 mg/kg) for 4 weeks only) with TC-5RW given as a single subcutaneous injection 30 days before prion inoculation. (d) Combination of rapamycin (treatment as in c) with 60SH-50 given as a single subcutaneous injection 30 days before prion inoculation. CEs were given at a dose of 4 g/kg body weight. Experiments were stopped at 250 dpi.

**Figure 4.**
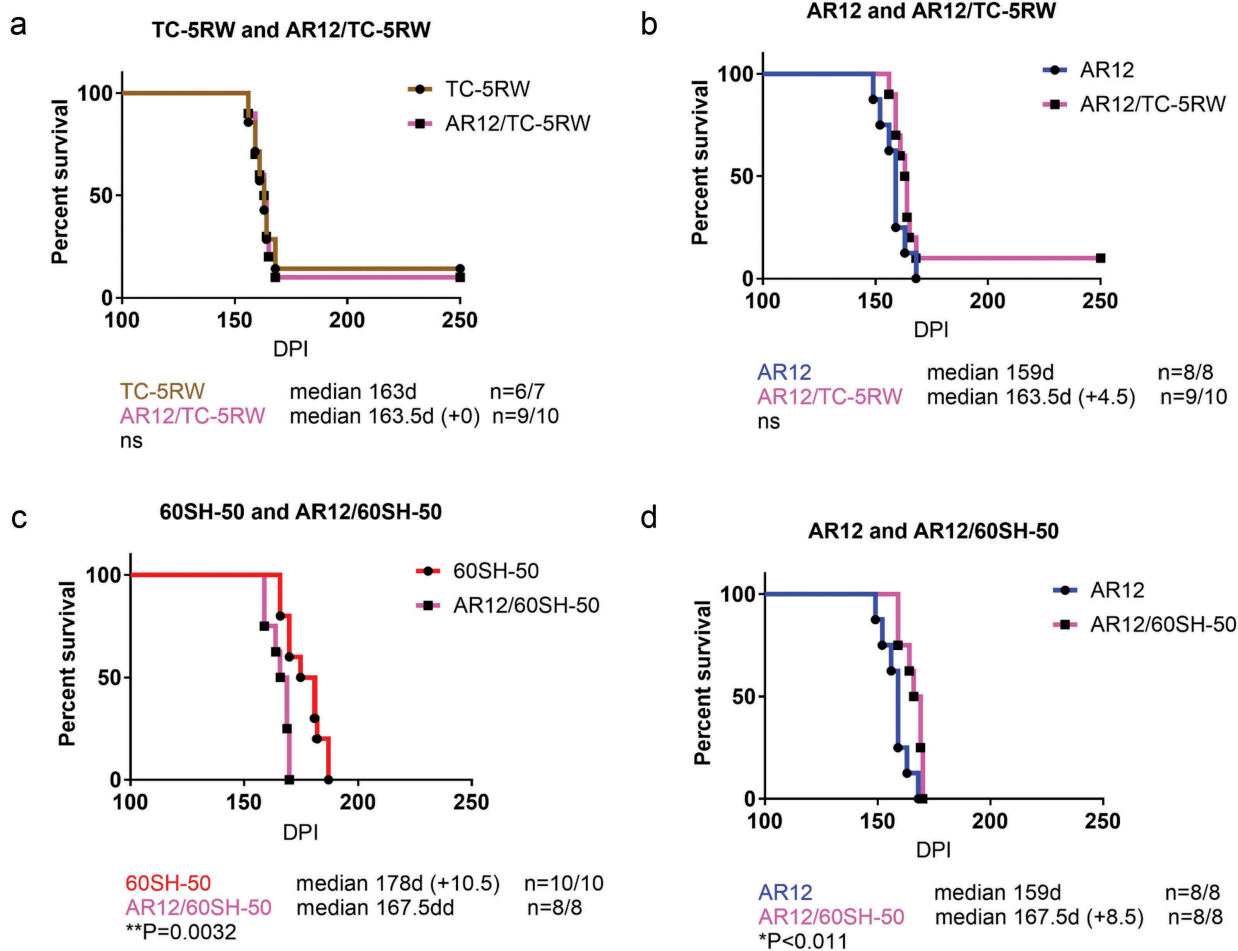
Combination of autophagy stimulator AR-12 and cellulose ethers. Survival analysis of FVB mice infected i.c. with RML prions. For combination therapy, the mice were treated with a single subcutaneous injection of either CE-5RW (a, b) or 60SH-50 (c, d) 30 days before prion inoculation. Thirty days after prion inoculation, the mice were treated with AR-12 as above, until the end of the experiment (250 dpi).

**Figure 5.**
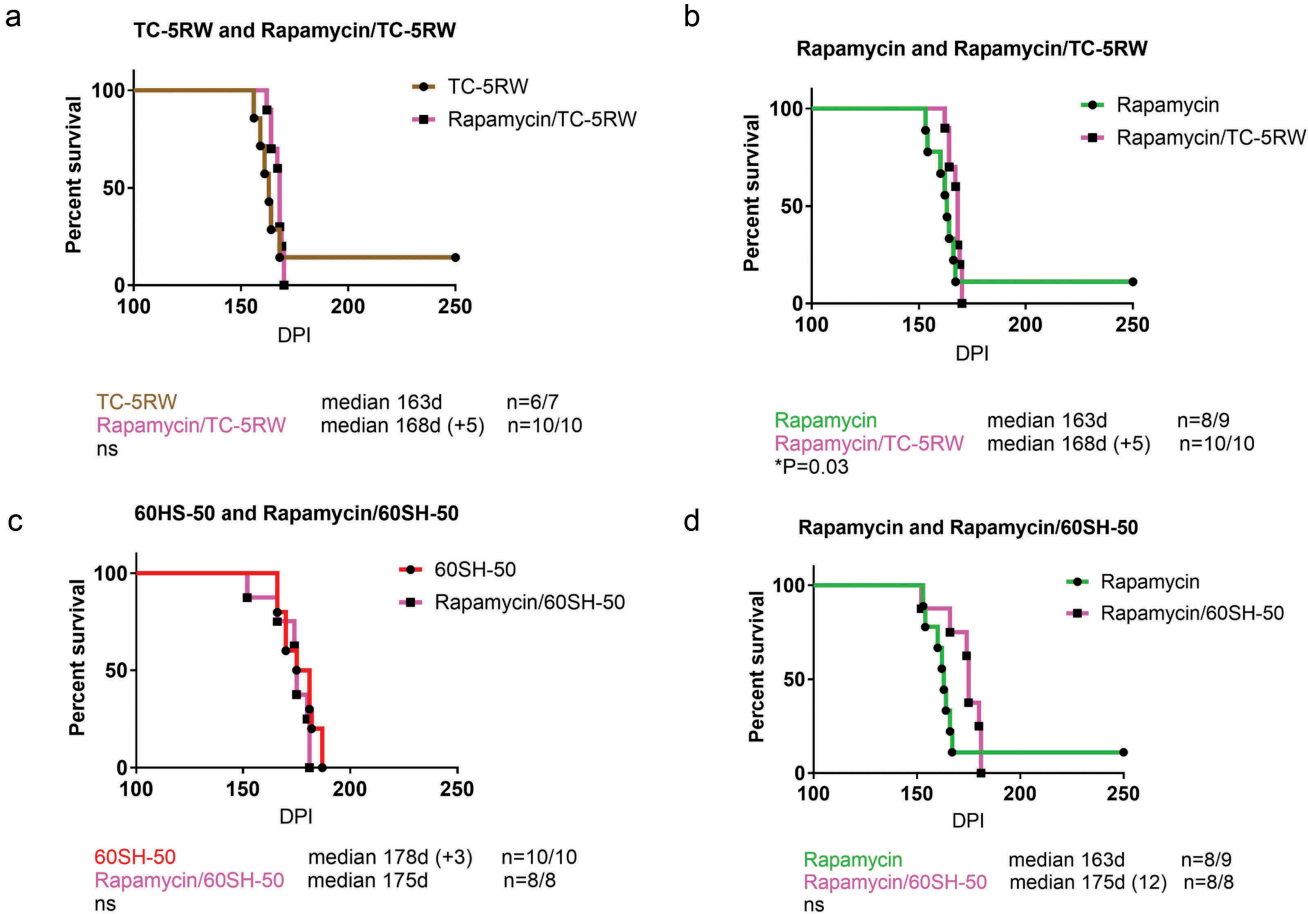
Combination of autophagy stimulator rapamycin and cellulose ethers. For combination therapy, the mice were treated with a single subcutaneous injection of either CE-5RW (a, b) or 60SH-50 (c, d) 30 days before prion inoculation. Thirty days after prion inoculation, the mice were treated with rapamycin as above (4 weeks). Experiments were stopped 250 dpi.

The promising outcome using the aforementioned compounds motivated us to test a combination therapy of autophagy stimulators and CEs. We had four groups of combinations, AR12 with TC-5RW, AR12 with 60SH-50, rapamycin with TC-5RW, and rapamycin with 60SH-50. Of note, the same regimen of treatment that has been used with the single drug treatments was applied for the combinations. CEs (TC-5RW or 60SH-50) were given as a single subcutaneous dose, 30 days before i.c. inoculation with RML prions. Thirty days after inoculation, animals received either AR12 or rapamycin twice per week (i.p.) for 4 weeks. After 4 weeks, AR12 treatment was continued until the experimental endpoint by applying the drug with the drinking water (Figure 1(c)). This was not possible for rapamycin due to its poor solubility in water. All mice treated with the drug combinations showed significantly extended survival times compared to the untreated group (Figure 3(a–d)). However, when we compared the survival times of mice co-treated with AR12 and TC-5RW to those treated with either of the drug alone, we did not find a significant difference (Figure 4(a,b)). Surprisingly, combining AR12 and 60SH-50 resulted in a significant decrease in the survival compared to the group treated only with 60SH-50 (*p = 0.0032*) (Figure 4(c)). Yet, there was a significant increase in the survival of the AR12 and 60SH-50 co-treated group compared to the AR12 only treated group (*p = 0.011*) (Figure 4(d)). Similar results were obtained with the combination of rapamycin and TC-5RW. While treatment with the drug combination did not result in a significantly different survival time compared to the TC-5RW treated group, a significant increase of the survival compared to the rapamycin only treated group was found (*p = 0.03*) (Figure 5(b)). Furthermore, comparing the combination of rapamycin with 60SH-50 to treatment with rapamycin only or 60SH-50 only, similar survival times were observed (Figure 5(c,d)).

Taken together, combination therapy resulted in prolonged survival compared to the untreated group. However, none of the combination groups showed better effects than 60SH-50 or TC-5RW treatment alone.

### Combining autophagy stimulators and cellulose ethers alleviates the autophagy-inducing activity of AR12 and rapamycin

**Figure 6.**
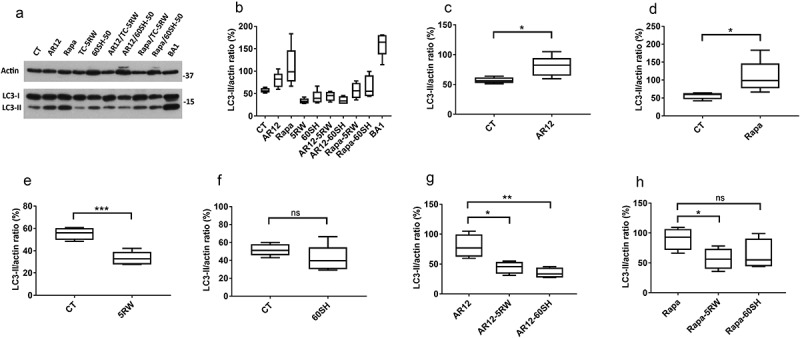
Impairment of the autophagy stimulatory effect of AR12 and rapamycin upon combining with cellulose ethers in N2a cells. (a) Neuronal cells (N2a) were treated with either AR12 (3 µM), rapamycin (500 nM), TC-5RW (3 mg/ml), 60SH-50 (3 mg/ml), bafilomycin A1 (100 nM), or DMSO (vehicle control) for 6 h. Cells were then lysed and LC3-I/II levels assessed in immunoblot analysis. Actin was used as a loading control. (b-h). Densitometry for panel A. LC3-II levels were normalized to actin and compared. *p = 0.05, **p = 0.01, ***p = 0.001, ns = non-significant. n = 5.

Our previous experiments have shown that treatment with either autophagy stimulators or CEs significantly increased the survival; however, the combination of those drugs did not result in an improvement of survival times. Rather, the survival times of mice treated with the drug combinations were similar to those treated only with CEs. Therefore, we hypothesized that CE treatment might negatively interfere with autophagy stimulation. To verify this, we treated cultured neuronal N2a cells with either AR12, rapamycin, TC-5RW, and 60SH-50 alone, or a combination of one autophagy stimulator with one of the CEs, and analysed autophagy stimulation using LC3-II levels as a readout. Treatment with the lysosomal inhibitor Bafilomycin A1 was used as positive control, resulting in the accumulation of LC3-II levels inside cells (Figure 6(a,b)). As expected, treatment with AR12 or rapamycin showed a significant increase in LC3-II levels (Figure 6(a,c,d)). Interestingly, we found that TC-5RW treatment significantly decreased the LC3-II levels (Figure 6(a,e)), however 60SH-50 did not (Figure 6(a,f)). Our results indicate that combining autophagy stimulators and cellulose ethers significantly decreased the LC3-II levels when compared to LC3-II levels in cells treated with AR12 or rapamycin alone (Figure 6(a,g,h)).

Taken together, our *in vitro* studies in cultured neuronal cells show a mitigation of the autophagy-inducing activity of AR12 and rapamycin upon combining them with cellulose ether compounds. This might explain the lack of additive or synergistic effects of the combinations in vivo.

## Discussion

Prion diseases are prototypic neurodegenerative disorders which manifest in humans as sporadic, genetic and acquired-by-infection forms, all being strictly fatal [2]. They come with an incidence of about one in a million worldwide. Although rare, about 6,000 to 7,000 individuals die every year from such diseases. The vast majority of human prion diseases are sporadic and have a clinical onset peak around 60 years of age, and rapidly progress when symptomatic [36]. Forms acquired by infection come with a distinct exposition risk to exogenous factors and can have epidemic character [37]. Classical examples are kuru infection, iatrogenic CJD and vCJD. Since prion diseases are characterized by a long incubation time followed by rapid clinical phase, therapy should be initiated before clinical symptoms manifest and before major damage is already present in the central nervous system. The human form with probably the longest incubation time is genetic prion disease. Here, a defined and disease-associated mutation is present from birth in the gene encoding the human prion protein [38]. Being a rare and fatal disease with a fast progression, and the absence of reliable preclinical markers dampened the search for therapeutic options. Only a few clinical trials were done, and mostly had outcomes which were not encouraging [39,40].

On the other hand, a huge variety of compounds with anti-prion properties or of other approaches targeting prion infection has been described [41,42]. Most of these compounds were established in cultured cells persistently infected with prions, using effects on prion propagation as main read-out. Cell death features usually are absent in such cell models. A significant proportion of such compounds were further validated in animal models of prion disease. Main read-out here is extension of incubation time to clinical disease, with describing an extension of 10–20% as already very successful and promising [25,42].

What are the reasons that the results obtained *in vitro* and in animal models are poor predictors for therapy in humans? First of all, these are strictly diseases of the central nervous system, and compounds have to pass the blood-brain barrier at concentrations which are effective, but not yet toxic. Of note, most anti-prion compounds described so far contain positive or negative charges and are less likely to effectively cross the blood-brain barrier [42,43]. Another hindrance is that *in vitro* and *in vivo* studies are usually done with mouse-adapted scrapie strains, which can react differently to therapeutic approaches than would do human prions [44]. Furthermore, long-term treatment could induce the development of resistant prions, as described previously [45]. Some of these obstacles could be addressed by combination therapy. As done for chemotherapy in infectious diseases and in cancer, a sophisticated combination of compounds which target different steps can achieve additive effects and reduce required concentrations of individual drugs and side effects.

Using this premise, we combined in this study compounds which seemed to address different molecular targets in prion pathogenesis. One group of substances covered inducers of autophagic activity to increase prion clearance (rapamycin and AR12 [25]), and was administered starting at day 30 after prion infection. These drugs were combined with cellulose ethers (TC-5RW and 60SH-50 [30]), which were applied as a single dose 30 days before infection. This combination seemed therefore less likely to be prone to drug interactions. As animal model, we used wild-type mice infected with RML prions, at a high dose and intracerebrally. We feel this is a model with aggressive disease development which well addresses a pre-clinical therapy scenario.

AR12 (OSU-03012) is a derivative of the anti-inflammatory compound celecoxib; however, it lacks the anti-inflammatory effect. AR12 was reported to have anti-cancer, antifungal and anti-microbial activity [46–49]. AR12 can induce autophagy activity [29,50,51]. We showed that treatment with AR12 enhanced the degradation of PrP^Sc^ and cleared prion seeding activity in cell line models infected with different prion strains [29]. Of note, AR12 has been reported to cross the blood-brain barrier effectively [52]. In the present study, we show an improved survival time of infected mice treated with AR12 compared with mock-treated controls. This is the first study which shows that AR12 has anti-prion effects *in vivo*.

The second autophagy stimulator was rapamycin. Rapamycin is a macrolide compound with immunosuppressant functions, which is used in humans for preventing the rejection of organ transplants [53,54]. It is a classical mTOR inhibitor and established stimulator of autophagy. Rapamycin was studied in various neurological and neurodegenerative disorders [55–59]. For prion diseases, we have shown that rapamycin treatment extended the survival of prion-infected mice when administered orally starting at day 100 post-infection [26]. Another study showed that rapamycin-treatment of Tg(PrP-A116V) mice, a model of Gerstmann-Sträussler-Scheinker syndrome, delayed disease onset and prolonged survival [35]. In the present study, we demonstrate that treatment with rapamycin for 4 weeks only extended the survival compared to the mock-treated group by 9%, corroborating the previous findings that rapamycin is a candidate for anti-prion therapy.

Over the last decades polymers showed up as drugs with anti-prion potential. Sulfated glycans [60,61], cationic dendrimers [62,63] and polyamines [64–66] have been used as anti-prion compounds. Recently, work from one of us identified cellulose ethers as a promising candidate. The most remarkable feature of CEs is that it is sufficient to administer a single dose, even long before prion infection occurs [30]. CEs do not have similarity in structure or chemical properties with previously described anti-prion polymers. Although the addition of TC-5RW inhibited 263K prion amplification *in vitro* in protein misfolding cyclic amplification reaction (PMCA), it is not clear yet how such polymers suppress prion disease *in vivo* and prolong the animal survival. Effects on autophagy were excluded [30]. Of note, the effectiveness of CEs was affected by the type of prion strain and animal model used [30].

In the current work, we speculated that combining CEs with stimulators of autophagy would result in additive effects. Unfortunately, this combination therapy did not result in a significant prolongation of incubation time when compared to mice treated with CEs alone. There are several reasons for this. One could be that the administration of autophagy inducers was sub-optimal, as being rather early in disease pathogenesis (day 30–60 p.i. applied i.p.). Only AR12 treatment was continued via drinking water, which might be less effective than i.p. application. For follow-up studies, drug application should be started later and should be prolonged. In addition, infecting the mice with a lower dose of prions might show protective effects better. Since we also had one combination treatment group which succumbed to disease earlier, we investigated the possibility that one drug group could antagonize the effect exerted by the other group. We focused on the well-defined autophagy-inducing effects of rapamycin and AR12, and performed a detailed *in vitro* analysis in cultured neuronal N2a cells. To our surprise, this showed that the autophagy-inducing activity of AR12 and rapamycin was neutralized upon combining them with CE compounds. Interestingly, TC-5RW application even resulted in a significant decrease of the levels of the autophagy marker LC3-II in N2a cells. This might provide an explanation of why our *in vivo* experiments did not work out as expected.

Although our trial failed to create additive effects, it illustrates both the need to empirically test promising drug combinations *in vivo*, and the requirement to exclude antagonistic mechanistic effects *in vitro*, if possible. The concept of establishing and validating appropriate combination therapies for prion diseases *in vivo* is valid, and there clearly will be a place for combination therapy in the future.

## Materials and methods

### Reagents

AR12 (also known as OSU-03012) was purchased from Medkoo Bioscience (200272). Rapamycin was purchased from LC Laboratories (R-5000), and Bafilomycin A1 from Sigma Aldrich (B1793). CE compounds TC-5RW and 60SH-50 were dissolved at a concentration of 5% in water. Compound properties and details are as previously described [30]. Anti-β-actin mAb was obtained from Sigma Aldrich (A5441) and anti-LC3 mAb (Clone 2G6) from NanoTools (0260–100). Peroxidase-conjugated immunoglobulins (goat anti-mouse HRP) was from Jackson Immuno-research Lab (115-035-003).

### Ethics statement

All animal experiments were performed strictly following the Canadian Council for Animal Care guidelines and were approved by the University of Calgary Health Sciences Animal Care Committee (protocol number AC18-0030 for CE treatment). The experiments involving the propagation of RML prions in FVB mice obtained from Charles River Laboratories were approved under protocol number AC14-0165.

### Drug treatment

Rapamycin was dissolved in 950 µl pure ethanol/50 µl DMSO as a 20 mg/ml stock solution and diluted in injection buffer (4% ethanol, 5% Tween 80, 5% PEG400 in dH_2_O) on the day of injection to a dose of 20 mg/kg in a final volume of 100 µl. Mice were injected twice per week. Control mice received a similar volume of injection buffer without active drug [67]. Treatment continued for 4 weeks. AR12 was dissolved in 950 µl pure ethanol/50 µl DMSO as a 5 mg/ml stock solution and diluted in injection buffer on the day of injection to a dose of 5 mg/kg in a final volume of 100 µl. Mice were injected twice per week. Control mice received a similar volume of injection buffer without active drug. Treatment continued for 4 weeks. Then, AR12 was given in drinking water at a concentration of 50 µg/ml until the end of the experiment. Cellulose ethers TC-5RW and 60SH-50 were applied as a single subcutaneous dose of 4 g/kg 30 days before prion inoculation.

### Mouse bioassay

Six-week-old female FVB mice obtained from Charles River Laboratories were treated with a single subcutaneous dose of cellulose ethers or left untreated. After 30 days, mice were inoculated under anaesthesia in the parietal lobe with 20 µl of 1% brain homogenate from terminally sick mice inoculated with prion strain RML, or with non-infected brain. For the intracerebral inoculation, 25-gauge disposable needles were used. After inoculation, mice were observed daily for any adverse conditions. Thirty days after prion inoculation, the animals were injected intraperitoneally with either rapamycin or AR12 for 4 weeks. Treatment with rapamycin was stopped and AR12 treatment was administered in drinking water until the end of the experiment. The animals were monitored for progression of clinical prion disease. At the terminal stage of disease, animals were sacrificed under anaesthesia, and the survival time of each animal was recorded.

### Maintenance of cell culture

The mouse neuroblastoma cell line N2a was obtained from ATCC (CCL-131) and cultured in Gibco OptiMEM Glutamax medium obtained from GIBCO, 51985–34 containing 10% fetal bovine serum obtained from Sigma Aldrich (F1051), and penicillin/streptomycin in a 5% CO_2_ atmosphere. N2a cells were plated overnight. The next day, cells were treated with either AR12 (3 µM), rapamycin (500 nM), TC-5RW (3 mg/ml), and 60SH-50 (3 mg/ml) alone, or a combination of one autophagy stimulator with one of the CEs. Bafilomycin A1 (100 nM) was used to block the lysosomal fusion resulting in pronounced accumulation of LC3-II. Drug treatment was done for 6 h followed by cell lysis.

### Cell lysis

N2a cells were lysed in cold lysis buffer (10 mM Tris-HCl, pH 7.5; 100 mM NaCl; 10 mM EDTA; 0.5% Triton X-100; 0.5% sodium deoxycholate (DOC)) for 10 min. Then, cell lysates were centrifuged to remove the cell debris followed by methanol precipitation. Lysates were frozen at −20°C until used for Western blotting.

### Western blotting

Methanol precipitated cell lysates were centrifuged, and pellets resuspended in TNE buffer (50 mM Tris-HCl pH 7.5; 150 mM NaCl; 5 mM EDTA). 12.5% SDS-PAGE was used. Gels were electro-blotted on Amersham Hybond P 0.45 PVDF membranes (Amersham, 10600023) and incubated with the desired antibodies. Luminata Western Chemiluminescent HRP Substrates from Millipore (WBLUF0100) was used for development. Densitometry was done using ImageJ program (NIH, USA). LC3-II signals were normalized to actin for quantification.

### Statistical analysis

Statistical analysis was performed using GraphPad Prism (GraphPad Software, version 7.03). For survival, the percent survival was plotted in a Kaplan–Meier plot, and a log-rank (Mantel–Cox) test was performed. Statistical significance for immunoblots was expressed as mean ± S.D. LC3-II levels were normalized to actin and compared using either the unpaired two-tailed *t-test* for pair-wise comparisons or the *One-way ANOVA* analysis with *Tukey post test* for multiple comparisons. *, p < 0.05; **, p < 0.01; ***, p < 0.001.
